# Supplementary material for: From Lab to Clinic and Farm: Leveraging Drosophila Feeding Studies to Combat Eating Disorders and Pest Challenges
Source: Biology (Basel). 2025 Sep 2;14(9):1168. doi: 10.3390/biology14091168 (PMC12467617; doi:10.3390/biology14091168)
Supplement: Supplementary file 1 [file biology-14-01168-s001.zip › biology-3723689-supplementary.pdf]

Supplementary Table S1. List of human genes associated with eating disorders (MalaCards) along with their *Drosophila* ortholog (if any) and DIOPT scores.

**Human Gene**	***Drosophila* Orthology**	**Orthology Score**
ADIPOQ	-	-
ADIPOR1	AdipoR	14/14
AGRP	-	-
ANKK1	Slpr, Tak1, Takl1	2/14
ANON1	Elite gene	-
BDNF	-	-
BULN	-	-
C1QL3	-	-
CCK	CCKLR-17D1, CCKLR-17D3	11/14
CCKAR	CCKLR-17D1, CCKLR-17D3	11/14
CNR1	-	-
CNR2	-	-
COMT	-	-
CRH	-	-
CRHR1	Dh44-R1, Dh44-R2	13/14
CRHR2	Dh44-R1	13/14
DPP4	CG11034	10/14
DRD2	Dop2R	8/14
DRD4	Dop2R	2/14
FAAH	CG5112, CG7900, CG7910	5/14
FTO	-	-
GAL	-	-
GCG	-	-
GH1	-	-
GHR	Cow	1/14
GHRH	-	-
GHRL	-	-
GHSR	CapaR, PK1-R, PK2-R1, PK2-R2	3/14
HCRT	-	-
HCRTR1	SIFaR	3/14
HTR1A	5-HT1A	11/14
HTR1B	5-HT1A	4/14
HTR2A	5-HT2B	9/14
HTR2C	5-HT2B	8/14
HTR3A	nAChRα1-4, nAChRβ1-3, NtR	2/14
IGF1	Ilp1, Ilp5	2/14
IGFBP1	-	-
IGFBP2	-	-
IGFBP3	-	-
INS	-	-
KCNN3	SK	12/14

LEP	-	-
LEPQTL1	-	-
LEPR	-	-
LOC110806262	-	-
MAOA	shps	4/14
MC3R	5-HT1B	1/14
MC4R	-	-
NPY	-	-
NR3C1	ERR	3/14
NTRK2	Nrk, otk, Ror	2/14
NTS	-	-
NUCB2	NUCB1	12/14
OPRM1	AstC-R2	5/14
OXT	-	-
OXTR	-	-
POMC	-	-
PPY	-	-
PRL	PRL-1	11/14
PYY	-	-
RETN	-	-
SHBG	CG11703	1/14
SLC6A3	DAT	12/14
SLC6A4	SerT	10/14
TNFRSF11B	-	-
TTR	CG30016	6/14
